# Deep learning based ultra‐low dose fan‐beam computed tomography image enhancement algorithm: Feasibility study in image quality for radiotherapy

**DOI:** 10.1002/acm2.14560

**Published:** 2024-11-14

**Authors:** Hua Jiang, Songbing Qin, Lecheng Jia, Ziquan Wei, Weiqi Xiong, Wentao Xu, Wei Gong, Wei Zhang, Liqin Yu

**Affiliations:** ^1^ Department of Radiation Oncology The First Affiliated Hospital of Soochow University Suzhou China; ^2^ Real Time Laboratory, Shenzhen United Imaging Research Institute of Innovative Medical Equipment Shenzhen China; ^3^ Radiotherapy Business Unit Shanghai United Imaging Healthcare Co. Ltd. Shanghai China; ^4^ Zhejiang Engineering Research Center for Innovation and Application of Intelligent Radiotherapy Technology Wenzhou China

**Keywords:** deep learning, fan‐beam CT, image quality, image‐guided radiotherapy, ultra‐low dose CT

## Abstract

**Objective:**

We investigated the feasibility of deep learning‐based ultra‐low dose kV‐fan‐beam computed tomography (kV‐FBCT) image enhancement algorithm for clinical application in abdominal and pelvic tumor radiotherapy.

**Methods:**

A total of 76 patients of abdominal and pelvic tumors were prospectively selected. The Catphan504 was acquired with the same conditions as the standard phantom test set. We used a CycleGAN‐based model for image enhancement. Normal dose CT (NDCT), ultra‐low dose CT (LDCT) and deep learning enhanced CT (DLR) were evaluated by subjective and objective analyses in terms of imaging quality, HU accuracy, and image signal‐to‐noise ratio (SNR).

**Results:**

The image noise of DLR was significantly reduced, and the contrast‐to‐noise ratio (CNR) was significantly improved compared to the LDCT. The most significant improvement was the acrylic which represented soft tissue in CNR from 1.89 to 3.37, improving by 76%, nearly approaching the NDCT, and in low‐density resolution from 7.64 to 12.6, improving by 64%. The spatial frequencies of MTF10 and MTF50 in DLR were 4.28 and 2.35 cycles/mm in DLR, respectively, which are higher than LDCT 3.87 and 2.12 cycles/mm, and even slightly higher than NDCT 4.15 and 2.28 cycles/mm. The accuracy and stability of HU values of DLR were similar to NDCT. The image quality evaluation of the two doctors agreed well with DLR and NDCT. A two‐by‐two comparison between groups showed that the differences in image scores of LDCT compared with NDCT and DLR were all statistically significant (*p* < 0.05), and the subjective scores of DLR were close to NDCT.

**Conclusion:**

The image quality of DLR was close to NDCT with reduced radiation dose, which can fully meet the needs of conventional image‐guided adaptive radiotherapy (ART) and achieve the quality requirements of clinical radiotherapy. The proposed method provided a technical basis for LDCT‐guided ART.

## 1 INTRODUCTION

Image‐guided radiotherapy (IGRT) plays an important role in clinical radiotherapy, especially for correction of postural errors during treatment, reduction of pose errors, improvement of pose reproducibility, and implementation of online adaptive radiotherapy (ART). It is helpful to reduce the uncertainty of anatomical changes, and improve the dosimetric metric of targets and OARs between different radiotherapy fractions.^1^, ^2^, ^3^, ^4^ In the abdominal and pelvic tumor, there are positional errors caused by changes in body position during different fractions. The targets and OARs are also susceptible to displacement and deformation by respiration, bladder filling, and intestinal peristalsis.^5^, ^6^, ^7^ Therefore, precise image guidance is needed.

The image guidance methods in clinical are cone beam CT (CBCT), magnetic resonance imaging (MRI), and ultrasound (US), CBCT is the most common image guidance method in clinical practice.^8^, ^9^, ^10^, ^11^, ^12^ However, the image quality of CBCT has more obvious noise and artifacts due to x‐ray scattering and motion^13^, ^14^ The IGRT usually relies on high‐density markers such as bone, while the low‐density contrast display is limited, such as soft tissue boundaries.^15^, ^16^ The above imaging guidance modalities make it difficult to operate precision radiotherapy in abdominal and pelvic tumors, such as stereotactic radiotherapy(SRT) and online ART in terms of imaging speed, imaging resolution, electron density information, and imaging quality. The integration of diagnostic kV‐FBCT with a conventional linear is a new modality for IGRT, which can provide diagnostic image during radiotherapy. FBCT can be directly used for OAR and target delineation and dose calculation for online ART.

Increasing the frequency of image guidance during radiotherapy can monitor the anatomical location and morphological changes of the tumor and OARs during treatment, thus improving the accuracy of radiotherapy. However, frequent use of CT scans can increase the radiation dose to the patients, which may lead to an increased risk of cancer, especially in children.^17^, ^18^ Therefore, we would like to reduce the radiation dose to a reasonably low and achievable level for the clinic while maintaining acceptable imaging quality.^19^ One direct method is to reduce the tube current during the CT scan. However, this LDCT imaging can significantly reduce image quality due to the limited number of photons detected.^20^ Currently, in order to maintain or improve the quality of CT images under low dose conditions, the usual methods of image processing are iterative image reconstruction (such as iDose4, IRIS, ASIR‐V, MBIR, IMR, etc.). Compared with the traditional filtered back projection (FBP) reconstruction algorithm, iterative reconstruction (IR) is more capable of reducing image noise, but too high a level of IR can alter the noise texture and greatly reduce the ability to diagnose small low‐contrast lesions at lower doses.^8^ Even more, the appearance or texture of the reconstructed images may be altered, and the spatial resolution remains unsatisfactory.^21^, ^22^, ^23^, ^24^, ^25^ It reduces the confidence of the physician. Currently, several deep learning (DL) based reconstruction algorithms have been applied to CT images and initially demonstrated strong image noise reduction,^9^, ^10^, ^11^ enabling further reduction of radiation dose and maintain image quality at the same time. In recent years, image reconstruction algorithm based on DL has been applied clinically to provide better images at low radiation doses.^26^, ^27^, ^28^, ^29^, ^30^ There are studies related to DL based algorithm in CBCT for image quality improvement.^31^, ^32^, ^33^, ^34^, ^35^, ^36^ however, there are fewer studies related to IGRT and ART for LDCT in the abdominal and pelvic radiotherapy^37^, ^38^ In this study, we proposed a CycleGAN ‐based DL model, which requires no paired training data and greatly reduces the difficulty of medical image data collection, was used as a common network structure for low‐dose CT image processing^27^, ^28^, ^29^ to train and learn low dose CT images guided by abdominal and pelvic tumor images and evaluate the quality of the trained low‐dose images can meet the further requirements of clinical radiotherapy/explore its generated clinical capability in low‐dose guidance of clinical tumor radiotherapy images. In this study, we propose a content noise cyclic consistent generation adversarial network (CNCycle‐GAN) based on CycleGAN to effectively decrease noise and artifacts from LDCT and improve image quality. The aim of this study focuses on HU accuracy, imaging signal‐to‐noise ratio (SNR) and imaging quality of LDCT enhanced by DL‐based algorithm can meet the requirements of clinical application, even online ART.

## 2 METHODS

### 2.1 Image collection

**FIGURE 1:**
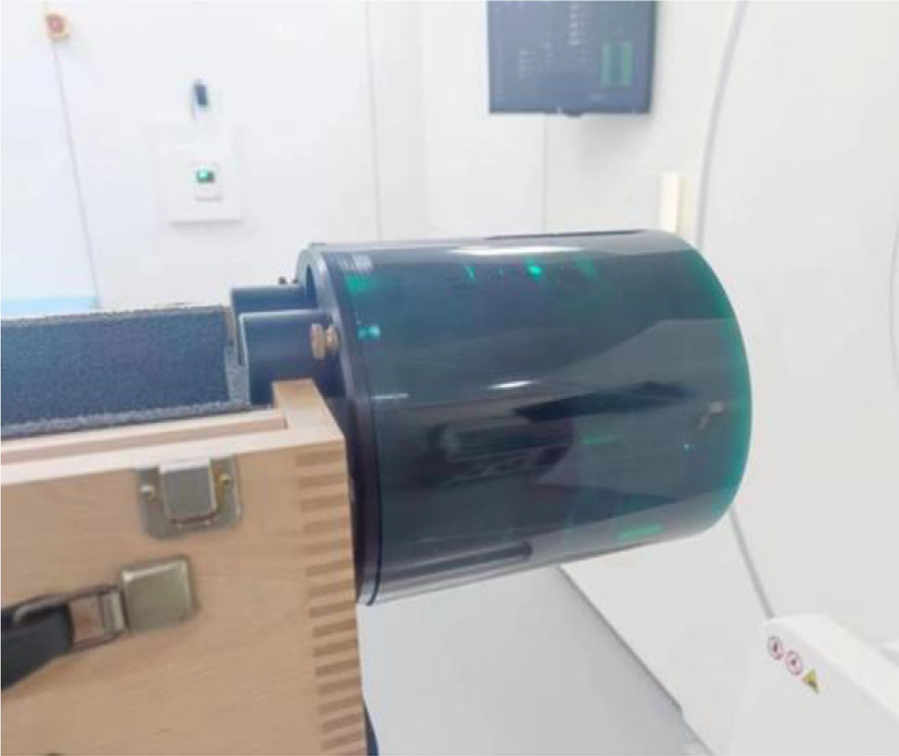
Catphan504 phantom.

Seventy‐six patients with abdominal and pelvic tumor treated at the Radiotherapy Center of the First Affiliated Hospital of Soochow University from January 2021 to May 2021 were prospectively selected to kV‐FBCT IGRT. The total patients included 26 males and 50 females. Patients aged 27 to 77 years old (58 ± 13.29 years old). Sixty‐eight cases were used for network training and the rest (8 cases) of them were used for testing. This was a prospective study approved by the local institutional review board, and all patients enrolled were informed and signed consent forms before treatment. The phantom was the Catphan 504 (Phantom Laboratory, USA), which was cylindrical with an internal diameter of 15 cm, an external diameter of 20 cm, and a length of approximately 25 cm.^5^ The embedded module CTP404 was approximately 4 cm thick and had two diagonal lines and eight cylindrical inserts embedded in the horizontal and vertical directions.^6^ The phantom contained seven small cylindrical samples of different substances with a diameter of 1.25 cm, four of which were selected for analysis: air, mimicking air cavity, low‐level polyethylene (LDPE), acrylic, mimicking the soft tissue and Teflon, mimicking the bone, as shown in Figure 1.

**FIGURE 2:**
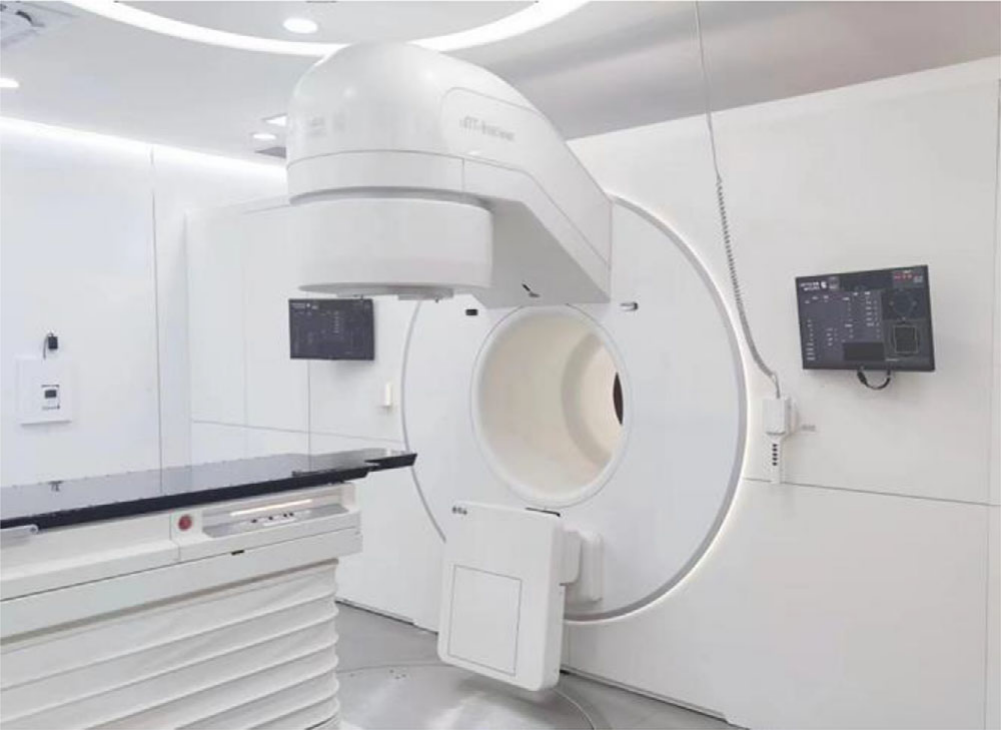
United Imaging uRT‐linac 506c.

The CT images were obtained using United Imaging uRT‐linac506c (Shanghai United Imaging Healthcare Co., Ltd, China), which was an integrated 16‐row diagnostic CT with an imaging aperture of 70 cm, shown in Figure 2. All patients were scanned in the supine position with hands raised on the forehead and fixed by a vacuum pad. Each patient was scanned with conventional dose and ultra‐low dose protocols to acquire images within the same fraction, which were recorded as NDCT and LDCT respectively. The scanning range was from sub diaphragm to umbilicus for abdominal tumors, and from L3 level to 5 cm below the sciatic node for pelvic tumors. Image scanning parameters: field of view (FOV) 50 cm, layer thickness 3 mm, layer spacing 3 mm, spiral pitch factor 0.9375, reconstruction matrix 512 × 512, reconstruction spatial resolution 0.9765 × 0.9765 mm^2^. The scanning tube voltage was 120 kV, and the standard tube current of 200 mAs was used for NDCT, while the standard tube current of 24 mAs was used for LDCT. The average dose length product (DLP) was 466.67 mGy * cm. Under low‐dose scanning conditions, the tube voltage was 120 kV and the tube current was 24 mA. The DLP was 44.67 mGy * cm. According to AAPM Report No.96, the factor of adult in abdominal and pelvic is 0.015 mSv/(mGy * cm). Therefore, the effective dose of the abdominal and pelvic cavity was 7.0 mSv under the condition of normal dose scanning, and the effective dose under the condition of low‐dose scanning was 0.67 mSv.

**FIGURE 3:**
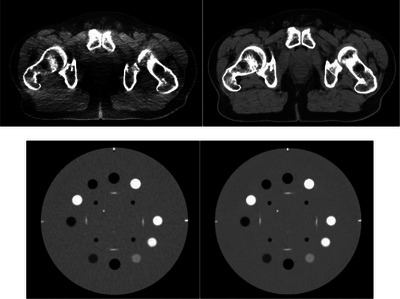
Comparison of NDCT (right) and LDCT (left).

For Catphan 504 scans, the phantom was suspended at the treatment bed and five sets of images were acquired for each protocol, with the same parameters for patient scans. The NDCT and LDCT of patients and phantom were shown in Figure 3. The reconstructed images were saved in DICOM format, and the reconstructed images were read and analyzed by a MATLAB program to calculate the image quality assessment parameters. The NDCT and LDCT were reconstructed using the system standard convolution kernel function, and uploaded to picture archiving and communication system (PACS) for analysis.

### 2.2 Network structure

**FIGURE 4:**
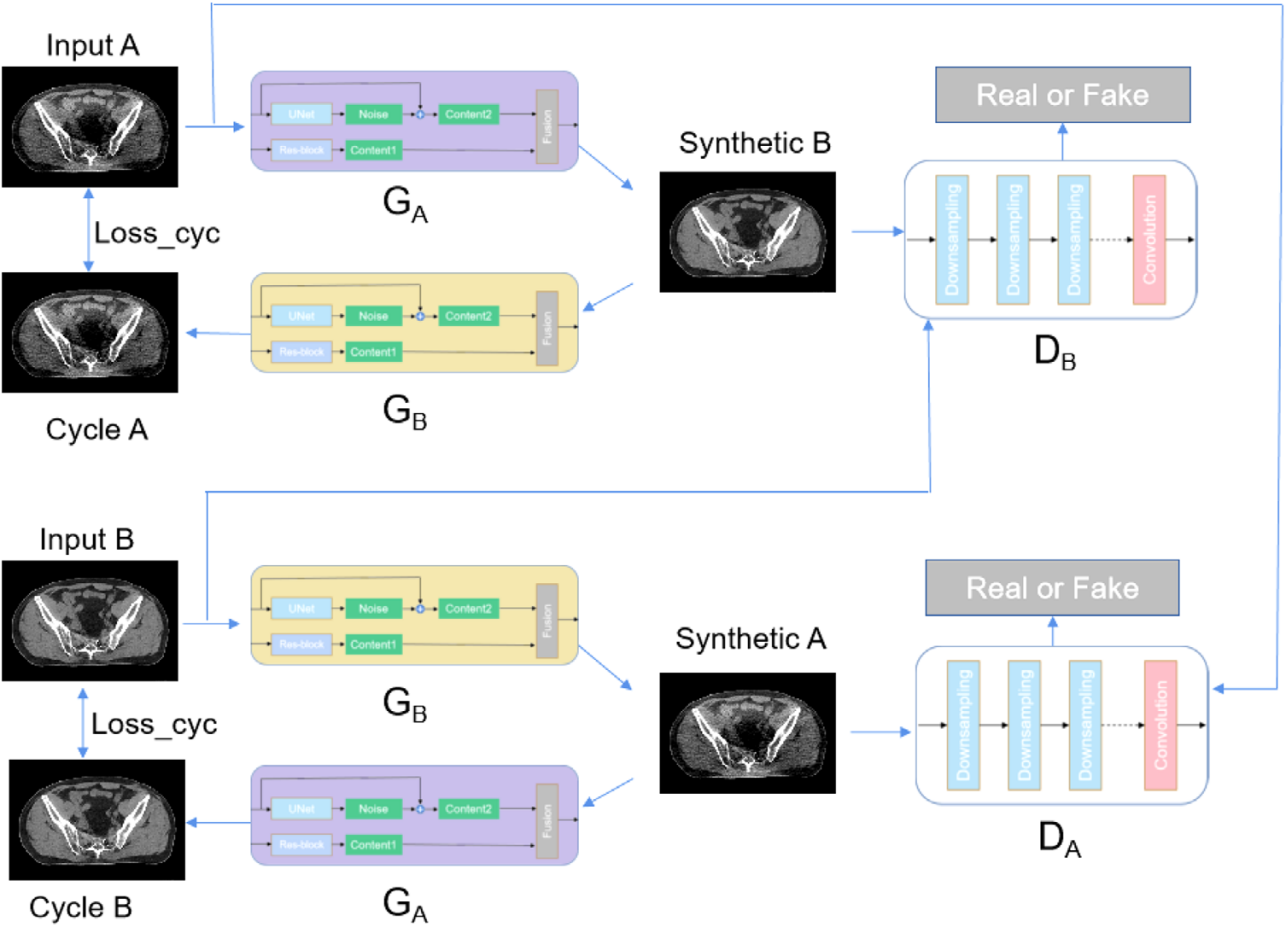
Network structure of CNCycleGAN.

As shown in Figure 4, we used CNCycle‐GAN network to implement LDCT enhancement, CNCycle‐GAN used CycleGAN as the basic network. Just like CycleGAN, CNCycleGAN used two generators and two discriminators for image translation between x domain and y domain. Unlike CycleGAN, CNCycle‐GAN uses two content‐noise convolutional networks as generators, that G_A_:A → B, which realizes the conversion of LDCT images to NDCT images; G_B_:B → A, which implements the conversion of NDCT to LDCT. Each generator is trained adversarial using the corresponding convolutional network discriminator D_B_ and D_A_.

For illustration purposes, the generator G_A_ input was the LDCT image I_A_ and the output was the generated NDCT image I_B_ = G_B_ (I_A_). The discriminator G_B_ input was the generated NDCT image and the real NDCT I_B_. These two networks G_A_ and D_B_ played each other in the training process, where D_B_ acted as a binary classifier to determine whether the input image was a real LDCT or not. On the other hand, the G_A_ serves to improve the fidelity of the generated NDCT and thus deceive the discriminator. The training process was formulated as an adversarial loss function $L_{adv}\left(G_{A},D_{B}\right)$:

(1) $$\begin{array}{ccc} \min_{G_{A}}\;\max_{D_{B}}L_{adv}\left(G_{A},D_{B}\right) & = & \log\left(D_{B}\left(I_{B}\right)\right)\\ & & +\log\left(1-D_{B}\left(G_{A}\left(I_{A}\right)\right)\right) \end{array}$$

Similarly, another set of generating adversarial losses $\;L_{cyc}\left(G_{A},G_{B}\right)$ can be expressed as:

(2) $$\begin{array}{ccc} \min_{G_{B}}\;\max_{D_{A}}L_{adv}\left(G_{B},D_{A}\right) & = & \log\left(D_{A}\left(I_{A}\right)\right)\\ & & +\log\left(1-D_{A}\left(G_{B}\left(I_{B}\right)\right)\right) \end{array}$$

We used cyclic consistent loss $L_{cyc}\left(G_{A},G_{B}\right)$ to ensure that a domain image was recovered as much as possible after the domain transformation of both generators, and to avoid direct interaction between the two domain images while constraining the generation direction of both generators for unsupervised network training.

(3) $$\begin{array}{ccc} L_{cyc}\;\left(G_{A},G_{B}\right) & = & ∥I_{A}-G_{B}\left(G_{A}\left(I_{A}\right)\right){∥}_{1}\\ & & +∥I_{B}-G_{A}\left(G_{B}\left(I_{B}\right)\right){∥}_{1} \end{array}$$

It had been noted that increasing the constant loss could improve the stability of the generator.^31^ Thus we also introduced the constant loss:

(4) $$L_{iden}\;\left(G_{A},G_{B}\right)=∥I_{B}-G_{A}\left(I_{B}\right){∥}_{1}+∥I_{A}-G_{B}\left(I_{A}\right){∥}_{1}$$

In summary, the total loss function of CNCycle‐GAN consisted of a weighted sum of the generative adversarial loss and the cyclic consistent loss, as shown in the following equation:

(5) $$\begin{array}{ccc} & & L\;\left(G_{A},G_{B},D_{A},D_{B}\right)=L_{adv}\;\left(G_{A},D_{B}\right)+L_{adv}\left(G_{B},D_{A}\right)\\ & & +\lambda_{cyc}L_{cyc}\left(G_{A},G_{B}\right)+\lambda_{iden}L_{iden}\left(G_{A},G_{B}\right) \end{array}$$

where $\lambda_{cyc}$ and $\lambda_{iden}$ were the weights used to control the importance of the corresponding losses.

### 2.3 Model training

Before training, the image data were pre‐processed including resampling to the same spacing, and central random cropping of the data to 256 × 256 image blocks. Statistical analysis of the image CT value distribution of the training set, then, Min‐Max normalization with −1000 and 90% of the maximum CT value. Most CT value information was retained. During the training process, data augmentation were used. In data augmentation, operations such as like flipping along the vertical direction, scaling and clip to a certain size, random clip and resize, and rotation with random degrees between 0° and 360° were applied without changing the value distribution of the image.

We used the ADAM optimization to train model by minimizing the loss function (5), where the $\lambda_{cyc}$ was 20, the $\lambda_{iden}$ was 0.5 and the number of epochs was 250. For the first 150 epochs, we set the learning rate to 0.0002 and reduce it linearly to zero in the next epochs. The batch‐size was 4. The kernel was randomly initialized from a Gaussian distribution. The proposed method was implemented using the PyTorch and used NVIDIA Quadro RTX 4000 to train. During testing, we used LDCT images from the test set as generator inputs to evaluate the image quality of DLR.

## 3 IMAGE QUALITY EVALUATION

### 3.1 Objective evaluation

The images were read and analyzed by MATLAB 2020a (The MathWorks, Inc., USA) program to calculate the image quality evaluation parameters, specifically: modulation transfer function (MTF),^2^, ^3^, ^4^, ^5^, ^6^ contrast to noise ratio (CNR),^3^, ^4^, ^5^, ^6^ low contrast visibility (LCV),^3^, ^4^, ^5^, ^6^ CT value uniformity (UI),^3^, ^4^, ^5^, ^6^ noise power spectrum (NPS),^7^, ^8^, ^9^ CT value mean absolute error (MAE), mean square error (MSE).^39^The formula to be used is as follows:

(6) $$\mathrm{CNR}=\frac{|\bar{HU}\left(material\right)\;-\;\bar{HU}\left(background\right)\;|}{\sqrt{\sigma {\left(material\right)}^{2}+\sigma {\left(background\right)}^{2}}}$$

(7) $$\mathrm{LCV}=2\frac{|\bar{HU}\left(LDPE\right)\;-\;\bar{HU}\left(PS\right)|}{\sigma \left(LDPE\right)+\sigma \left(PS\right)}$$

(8) $$\mathrm{UI}=\frac{|\bar{HU}\left(periphery\right)\;-\;\bar{HU}\left(center\right)|}{\bar{HU}\left(center\right)+1000}$$

(9) $$NPS\;\left(u,v\right)=\frac{x_{0}y_{0}}{N_{x}N_{y}}\;{\left|\sum_{i,j}\Delta g\left(i,j\right)*e^{-2\pi i\left(u*i*x_{0}+v*j*y_{0}\right)}\right|}^{2}$$

(10) $$MAE=\frac{1}{n}\;\sum_{i=1}^{n}\left|p_{i}-o_{i}\right|$$

(11) $$MSE=\frac{1}{n}\;\sum_{i=1}^{n}{\left(p_{i}-o_{i}\right)}^{2}$$

### 3.2 Subjective evaluation

Subjective scoring of CT image noise and outline of structures (targets and OARs) were performed by two senior physicians (S.Q. and L.Y, both of them have more than 20 years of experience in radiotherapy.) using a double‐blind method according to ART requirements respectively. Scores were used the Likert 5‐point scale method,^40^, ^41^ which was shown in Table 1.

**TABLE 1:** Subjective evaluation for image quality.

Score	Image noise	Clinical outline description
5/Excellent	No noise	Clear anatomical structure, clear images, clear boundaries, fully compliant with clinical requirements
4/Good	Minor noise	Clear anatomical structure, moderately blurred images, blur boundaries, compliant with clinical requirements
3/Medium	Medium noise	Anatomical structure is not clear, clear images, mildly blur boundaries, and some difficulty in outlining
2/Poor	Loud noise	Roughly identified images, unclear boundaries, not satisfied with the outline requirements
1/Bad	Severe noise	Image is blurred and cannot be used for outlining

If the image quality was between the two degrees, the score was based on the lower criterion, and two sets of scores were recorded.

### 3.3 Statistical analysis

SPSS 19.0 software (IBM, USA) was used for statistical analysis. The measures were assessed for normality using the Shapiro–Wilk test and were expressed as $\bar{X}$± s if they conformed to a normal distribution and as M (Q _1_, Q_3_) if they did not conform to a normal distribution. The Kruskal–Wallis nonparametric test was used to analyze the differences in objective ratings and subjective scores of the images between the two groups, with an adjusted *p*‐value of 0.017. The Cohen's Kappa method was used to do a consistency analysis of the subjective scores of the two physicians, and Kappa > 0.8 was considered very good consistency; 0.6 < Kappa ≤ 0.8 was considered good consistency; 0.4 > Kappa was considered a statistically significant difference.

## 4 RESULTS

### 4.1 Objective evaluation analysis

A comparison of MTF, LCV, and UI metrics for different images is shown in Table 2.

**TABLE 2:** Comparison of MTF, LCV, and UI of LDCT, DLR, and NDCT.

	LDCT	*P*‐value	DLR	*P*‐value	NDCT	*P*‐value
MTF10	3.29 ± 0.11	–	3.37 ± 0.28	>0.05	4.75 ± 0.85	>0.05
MTF50	1.81 ± 0.06	–	1.85 ± 0.15	>0.05	2.61 ± 0.46	>0.05
LCV	6.63 ± 0.23	–	10.59 ± 0.11	<0.05	14.82 ± 0.50	<0.05
UI (%)	0.17 ± 0.07	–	0.10 ± 0.04	>0.05	0.15 ± 0.00	>0.05

MTF reflected the ability of the image to distinguish high‐density but fine objects. MTF10 was closest to the subjective judgment of human. MTF of different images is shown in Table 2. As shown in Table 2, DLR get better MTF10 and MTF 50 than those of LDCT, which indicates that DLR had an improvement in the Imaging of high‐density fine objects compared with LDCT. However, the MTF of DLR was worse than NDCT. LCV was particularly important for the soft tissue display, and the larger the LCV value is, the stronger the ability to distinguish the density close to the tissue. DLR can suppress the noise more effectively, thus improving the LCV value. It improved from 6.63 to 10.59, an improvement of 59.7%, which will greatly improve the soft tissue resolution and tissue boundary. UI responded to the uniformity of CT value. DLR not only had significant noise suppression, UI was substantially improved over LDCT, even better than NDCT.

CNR shows the performance of visible low‐contrast objects. Larger CNR values indicate a better ability to distinguish target tissues from the background. Table 3 shows the results of CNR compared with LDCT, DLR, and NDCT. Compared with the LDCT, the CNR of the modalities of mostly different densities of tissues in the RCT images improved significantly after DL, with the modalities of Acrylic representing typical soft tissue density improving the most significantly, from 1.98 to 3.36, an improvement of 76%, which is close to NDCT. Among the above tissues, only the CNR of Teflon representing human bones has decreased, which is due to the reason for this is that when the CT images were pre‐processed to do normalization, the nonlinear normalization method of Min‐Max was used. When the CT values were too large, their CT values could not be recovered accurately. However, this study protocol was still able to recover most of the CT values of the bones without much impact on imaging, outlining, and dose calculation.

**TABLE 3:** Comparison of CNR for LDCT, DLR, and NDCT.

	LDCT	*P*‐value	DLR	*P*‐value	NDCT	*P*‐value
Air	78.99 ± 1.84	–	116.35 ± 2.70	<0.05	180.58 ± 4.14	<0.05
PMP	22.46 ± 1.61	–	34.73 ± 2.02	<0.05	53.59 ± 1.04	<0.05
LDPE	14.81 ± 0.54	–	25.43 ± 0.45	<0.05	36.14 ± 1.71	<0.05
Polystyrene	11.59 ± 0.33	–	21.08 ± 1.27	<0.05	22.94 ± 1.72	>0.05
Acrylic	1.98 ± 0.21	–	3.36 ± 0.50	<0.05	3.95 ± 0.25	>0.05
Delrin	17.96 ± 0.53	–	26.45 ± 1.01	<0.05	40.13 ± 0.97	<0.05

**FIGURE 5:**
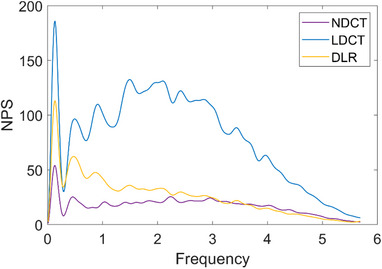
Comparison of NPS for NDCT, DLR, and LDCT.

Figure 5 shows the NPS of NDCT, DLR, and LDCT. The larger the curve amplitude indicates the greater the noise intensity at that spatial frequency. LDCT has a larger peak in the spatial low‐frequency region, indicating a larger difference in CT values at spatially distant locations, that is, a deviation in CT value uniformity, while DLR has a relatively small difference in CT values at spatially distant locations, also the closer to NDCT uniformity.

Table 4 compares the differences in CT values of the three images within the full‐mode and high‐density ROI. With the whole‐mode statistics, the differences in CT values were smaller for DLR, indicating less noise and good HU homogeneity; with the high‐density ROI statistics, DLR had some differences compared to LDCT, which was consistent with the CNR index.

**TABLE 4:** Comparison of MAE of CT values of LDCT and DLR.

	LDCT	DLR
Whole model	8.018 ± 0.065	6.116 ± 0.156
High density ROI	20.073 ± 0.575	25.386 ± 0.013

### 4.2 Subjective evaluation analysis

**FIGURE 6:**
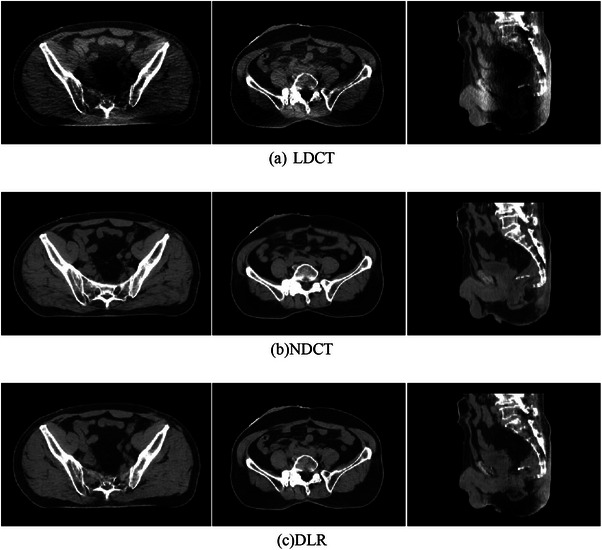
Comparison of LDCT, NDCT, and DLR.

The subjective evaluation of LDCT, DLR, and NDCT was shown in Figure 6. There are severe artifacts and noise in the center of the LDCT. As for DLR, the image quality was improved, close to NDCT. As shown in Table 5, a two‐by‐two comparison between groups showed that the differences between LDCT images and NDCT image scores in subjective noise and display of major outlined anatomical structures in GTVnx, GTVnd, CTV, rectum, small intestine, bladder, ovary (female), spinal cord, and femoral head were statistically significant (*H* = −7.096, −6.835, −7.198, −7.231, −7.156, −6.661, −7.156, −7.198, −7.124, −4.274, *p* < 0.001); the differences between LDCT and RCT image scores were statistically significant (*H* = −8.252, 8.299, 8.370, 8.229, 8.321, 8.4447, 8.321, 8.370, 8.283, 5.698, *p* < 0.001); the differences in subjective noise scores for both DLR and NDCT images were not statistically significant (*H* = 1.155, 0.176, 1.172, 0.997, 1.165, 1.787, 1.165, 1.172 1.160, 1.425, *p* > 0.05).

**TABLE 5:** Comparison of subjective scores of the three groups of images of six patients [M (Q_1,_ Q_3_)].

		
		Target			OARs					
Group	Image noise	GTVnx	GTVnd	CTV	Rectum	Small intestine	Bladder	Ovary (Female)	Spinal cord	Femoral heads
NDCT	5(5, 5)	5(5, 5)	5(5, 5)	5(5, 5)	5(5, 5)	5(5, 5)	5(5, 5)	5(5, 5)	5(5, 5)	5(5, 5)
LDCT	2(2, 2)	2(2, 2)	2(1, 2)	2(2, 2)	2(2, 3)	2(2, 2)	2(1, 2)	2(1, 2)	3(2, 3)	4(4, 5)
DLR	5(5, 5)	5(4, 5)	5(4, 5)	4(4, 5)	5(4, 5)	5(4, 5)	5(4, 5)	5(4, 5)	5(5, 5)	5(5, 5)
H value	82.191	83.847	85.022	78.755	80.115	76.555	78.337	86.674	83.330	74.125
*P*‐value	<0.001	<0.001	<0.001	<0.001	<0.001	<0.001	<0.001	<0.001	<0.001	<0.001

## 5 DISCUSSION

In this study, the noise reduction capability, image quality, and clinical application prospects of the DL‐based ultra‐low‐dose kV‐FBCT image enhancement algorithm in abdominal and pelvic tumor‐guided radiotherapy were evaluated by comparing the images with those of standard dose NDCT. CT dose reduction can be achieved by changing scanning parameters such as tube current (mA), pitch, tube potential/voltage (kVp) and rotation/exposure time.^42^ Conventional CBCT scans have a single dose of 1 to 2 cGy, with a maximum dose of 3.5 cGy.^43^, ^44^, ^45^ As the dose of radiation decreases, the number of photons must decrease, and when fewer x‐ray photons reach the detector, the image noise increases. will then increase^46^ The sharpness of image edges and boundaries will become blurred or even indistinguishable as the dose decreases, which affects the judgment of image guidance on the location of that target area and surrounding tissues and organs, and the accuracy of the target area outline or automatic image segmentation. There are more soft tissues in the abdomen and pelvis, and the difference in density values between adjacent tissues and tissues is relatively small, and gas and contents of the gastrointestinal tract and respiratory motion will increase the impact on image quality. There have been more studies showing that DLR algorithms for image reconstruction by DL can provide better image quality and lower image noise for low‐dose CT. Several scholars have used DL CycleGAN‐based algorithms to improve conventional CBCT image quality for ART studies feasibility^36^, ^47^, ^48^, ^49^, ^50^, ^51^ demonstrated that DLR technique improved image noise, overall image quality, CNR, and SNR of abdominal CT images by comparing with hybrid IR images.^52^ Compared the image quality of DLR and conventional IR in sub‐millisievert dose chest and pelvic abdominal CT. They found that the image quality of low‐dose CT with DLR was comparable to that of standard‐dose CT with IR. Lyu and Jensen et al.^53^, ^54^ detected low‐contrast liver metastatic lesions by DL image reconstruction at 50% and 75% radiation dose reduction, respectively, and found to maintain image quality comparable to that of full‐dose FBP and IR. Our results are similar to their findings, but the difference is that we performed a 90% reduction in radiation dose for image quality and to carry out an adaptation‐related basis, which presents a higher challenge. In terms of high contrast spatial resolution, both 10% MTF and 50% MTF indexes of RCT were higher than LDCT through phantom evaluation, and it has been shown that reconstruction enhancement algorithms that improve or preserve spatial frequency (tiny details) are beneficial for some high‐density tissues or enhance clearer boundaries such as blood vessels, while in tumor radiotherapy can enhance bony The sharpness of the edges of structures is beneficial for some patients with head and neck tumors and bone tumors that depend on bony localization. Online adaptation requires real‐time outlining of target volume and OAR as a prerequisite for ART, while abdominal and pelvic tumors have more soft tissue structures, and LCV is very important for the accuracy of radiotherapy OAR and target area boundary determination, target area outlining and automatic segmentation, and noise is bound to increase after radiation dose reduction, while LCV is inversely proportional to noise size is inversely proportional to the noise size, and when the noise is large, the useful signal will be drowned in the noise leading to a reduction in density resolution. Our results show that the DLR after DL can suppress the noise more effectively, and the LCV value improves from 6.63 to 10.59, an improvement of 59.7%. The CNR of most of the different tissue density modalities in RCT images was significantly improved, among which Acrylic representing typical soft tissue density had the most significant improvement, from 1.98 to 3.36, an improvement of 76%, which is close to NDCT. Localization of target areas and description of contours. The homogeneity and accuracy of CT values in ART studies are particularly important for the accuracy of planned dose calculations, and body model studies can be used to determine the homogeneity and validity of CT values for a single reconstruction technique.^55^ The UI reflects the CT value homogeneity of modality imaging, and DLR not only has higher imaging CT accuracy and significant noise suppression, the UI is substantially improved over LDCT and even better than NDCT. In abdominal and pelvic CT images, high image noise may mask subtle low‐contrast lesions in parenchymal organs, increasing the risk of missing similar liver tumors in the clinic as well as the risk of missed outline of target areas. In this study, we showed that LDCT had higher image noise and the lowest CNR on objective standard modality images, and we observed that the CNR of DLR images was significantly higher after reconstruction by DLR, showing better overall image quality, and there was no statistically significant difference between images comparing DLR and conventional dose CT data, and ultra‐low dose (20 mAs) CT images were also able to maintain low noise level, supporting the idea that our DL model can be used in further exploring the ability of lower dose images to guide radiotherapy, which is what we would like to see. We observed that the NPS curve amplitude in the LDCT group changed more, and the peak of the NPS curve was inversely proportional to the dose, the peak of the NPS curve in LDCT (24 mAs) was higher than 232 mAs, and there was a larger peak in the low‐frequency region, while the peak of the NPS curve in RCT under the same dose condition became smaller. This indicates that the CT value uniformity of DLR images is greatly improved after the DL image enhancement algorithm, and the fluctuation amplitude is smaller compared with LDCT, indicating less noise. The frequency corresponding to the peak of the curve hardly shifts due to the change of dose, which means that DLR reduces the image noise without changing the noise texture structure, which is consistent with the findings of Greffier, Li, and Nagayama.^29^, ^56^, ^57^ The NPS curve values of RCT images gradually shifted toward high frequencies with the enhancement of the reconstruction algorithm and became closer to NDCT, indicating the deepening of image protruding edges and the improvement of non‐correlation between pixel points, reflecting the improvement of image spatial resolution as well as detail contour legibility under the enhancement algorithm.^8^, ^9^ The main goal of LDCT image denoising is to recover the signal and noise texture present in NDCT images.^46^ In subjective scoring, the subjective observations of the two physicians were in high agreement. We found that due to the low scan dose, the boundaries of the tissues in the bladder region and rectum of the LDCT images were no longer discernible, and the pelvic region showed dark areas that could not be identified by the naked eye, with poorly delineated soft tissues and irregular radiolucent artifacts around them, while the RCT images generated after CycleGAN learning maintained a low image noise while comparing with NDCT for complete recovery of and preserved the original tissue structure, indicating that the DL method can reduce noise in addition to its loss function of finding the original information by fighting against the loss of image texture details and similarity, and that fighting against loss can prevent aggressive noise reduction and help retain fine details.^58^ The similarity index between DLR and NDCT bladder DSC reached 95%, and less image noise and artifacts were observed. However, smaller lymph nodes and blood vessels were also found to have less smooth edges, and some of the smaller (<0.5 cm diameter) low‐contrast lesions had blurred or even undetectable borders, which is consistent with Jensen et al.^54^, ^59^ This is consistent with the observation of Jensen et al. This point also reminds us that for image recovery and enhancement using DLR under ultra‐low dose conditions, the detectability of small lesions or tissues needs to be examined carefully to prevent any omission, which may require more targeted training of this type of lesion or tissue model in future DL models to improve the amount and coverage of data for each disease type.

The study is more accurate in extracting LDCT quantification through subjective and objective evaluation by patients and standard modalities, as opposed to patient tissue and organ evaluation, to identify details that need improvement and subjective selection of uncertainty at the ROI level. Also, patient‐based subjective evaluation can be more consistent with clinical applications, and patients undergoing radiotherapy for abdominal and pelvic tumors will benefit from maintaining high‐quality FBCT images at ultra‐low imaging doses, which in clinical practice will improve visual detection of soft tissues necessary for accurate visualization and target localization in IGRT. The improved soft tissue contrast will enhance localization including boundary contours, dose calculation and deformable image alignment, providing an important foundation for online adaptive radiation therapy for abdominal and pelvic tumors.

Limitations of our study: This study also needs to examine the recovery of DLR in more small low‐contrast lesions (<1 cm) at ultra‐low doses, which is what we need to investigate next. Since only typical abdominal and pelvic tumor patients were selected in our study, we did not evaluate the optimal combination of DLR at different sites as well as under different dose conditions and with different intensities. Future studies must provide a comprehensive understanding of the use of these reconstruction algorithms in oncology radiation therapy.

## 6 CONCLUSION

In this research work, DL based image enhancement algorithm improves the image quality of LDCT, which can maintain the subjective and objective image quality comparable to the standard dose NDCT used in conventional radiotherapy, and can be applied in clinical IGRT, and also provides a basis for the development of online ART for low‐dose FBCT‐based abdominal and pelvic tumors, which is worthy of clinical application and further exploration.

## AUTHOR CONTRIBUTIONS

Conception and design, acquisition of data: Hua Jiang, Lecheng Jia, and Ziquan Wei. Analysis and interpretation of data: Weiqi Xiong. Methodology and Software: Ziquan Wei, Weiqi Xiong, and Lecheng Jia. Validation and Investigation: Songbing Qin, Wei Gong, and Wentao Xu. Drafting the article or critical: Hua Jiang and Liqin Yu. Final approval of the manuscript: Linqin Yu. Supervision and resources: Songbing Qin and Wei Zhang.

## CONFLICT OF INTEREST STATEMENT

The authors declare no conflicts of interest.

## ETHICS STATEMENT

Ethical approval: “All procedures performed in studies involving human participants were in accordance with the ethical standards of the institutional and/or national research committee and with the 1964 Helsinki declaration and its later amendments or comparable ethical standards.” Informed consent: “Informed consent was obtained from all individual participants included in the study.”
